# Acceptability of hair harvest as a method of tuberculosis therapeutic drug monitoring among adult pulmonary TB patients: a qualitative study

**DOI:** 10.4314/ahs.v23i4.4

**Published:** 2023-12

**Authors:** Grace Muzanyi, David K Mafigiri, Robert Salata, Moses Joloba, Jackson Mukonzo, Mohammed Ntale, Paul Mubiri, Godfrey Bbosa

**Affiliations:** 1 Department of Pharmacology and Therapeutics, College of Health Sciences, Makerere University; 2 Uganda-Case Western Reserve University Research Collaboration; 3 Makerere University School of Public Health; 4 Case Western Reserve University, Cleveland Ohio; 5 Makerere University School of Social Sciences; 6 Makerere University School of Biomedical Sciences; 7 College of Natural Sciences, Makerere University

**Keywords:** Tuberculosis, hair harvest, hair testing, adherence monitoring, culture, faith

## Abstract

**Background:**

The current six months regimen for drug-susceptible tuberculosis (TB) is long, complex, and requires adherence monitoring. TB hair drug level assay is one innovative approach to monitor TB treatment adherence however, its acceptability in the context of African multi-cultural settings is not known.

**Objective:**

To determine the acceptability of hair harvest and testing as a TB therapeutic drug monitoring method.

**Methods:**

The study explored perceptions, and lived experiences among TB patients with regard to using hair harvest and testing as a method of tuberculosis therapeutic drug monitoring in the context of their cultural beliefs, and faith. We used a descriptive phenomenological approach.

**Results:**

Four main themes emerged namely: participants' perceptions about the cultural meaning of their body parts; perceptions about hair having any medical value or meaning; perceptions about hospitals starting to use hair harvest and testing for routine hospital TB treatment adherence monitoring; and perceived advantages and disadvantages of using hair for treatment adherence monitoring. Overall, we found that using hair to monitor adherence was acceptable to TB patients provided the hair was harvested and tested by a medical worker.

**Conclusion:**

Hair harvest for medical testing is acceptable to TB patients on the condition that it is conducted by a medical worker.

## Introduction

Tuberculosis (TB) still remains a global health problem^1^. The current six-month treatment regimen for drug-susceptible TB is complex, long, and associated with treatment default and the emergence of drug resistance. Recently the World Health Organization (WHO) announced a new four-month TB regimen for drug-susceptible TB^2^ but this is yet to be rolled out into the TB treatment policy of most countries around the globe. Given the current long duration of TB treatment and the associated high rates of default compounded with the emergence of drug resistance, it warrants TB treatment adherence monitoring to mitigate treatment default and the emergence of drug resistance.

Blood^3^,^4^, saliva^5^, and urine^6^ drug levels can potentially be used to monitor TB treatment adherence but all require daily sampling which is not convenient for the health care provider and the patients.

In HIV treatment, currently, hair drug levels of antiretroviral drugs are being used in some parts of the world to monitor HIV treatment adherence^7^,^8^,^9^,^10^, drug exposure, and treatment outcomes. However, hair harvest and testing for therapeutic drug monitoring are new in the African setting. Given the cultural and faith complexity of the African society^11^, ^12^ and the witchcraft beliefs attached to certain body parts, one needs to assess whether the use of hair drug assays in a routine hospital setting is acceptable to the patient communities.

## Methods

### Study design

We used a descriptive phenomenological approach to explore the acceptability of hair harvest as a method of therapeutic drug monitoring among pulmonary TB patients in the context of their faith, culture, and lived experiences.

### Study setting

This study was carried out at the Uganda Case Western Reserve University Research Collaboration TB project clinic within Mulago Hospital, Kampala, Uganda's largest national referral hospital. The TB project clinic is a specialized TB+HIV research clinic where patients residing within a radius of 30km are screened and enrolled. The catchment area includes places like the high TB endemic five divisions of Kampala District 13.

### Participants and recruitment

Participants were confirmed (Genexpert+/smear+) TB patients who had already started TB treatment. Participants were recruited by the Principal Investigator (PI) through screening at the local TB clinic at the Uganda-Case Western Reserve University research collaboration. The inclusion criteria were confirmed TB patients aged at least 18 years who had started TB treatment. The exclusion criteria included TB patients who wre below 18 years of age and those with no TB treatment experience. A purposive sampling strategy^14^ was used to recruit 16 TB patients who were able to provide in-depth information regarding the use of hair harvest and testing as a method for TB therapeutic drug monitoring in routine hospital care. This sampling strategy was used in this phenomenological approach as it allows the selection of TB patients with a rich source of knowledge.

### Data collection

All interviews were conducted in person and audio recorded. The interviews were conducted in Luganda, the local language most widely spoken in the study catchment area. The Luganda language interviews were then transcribed by the interviewer into MS Word. The Luganda transcript was then translated into English by an independent research assistant. The English transcript was then back-translated into Luganda by the interviewer who had conducted the original interview. Any differences in opinion or areas that needed consensus about meaning and representation were discussed and reviewed together with the PI. Data collection continued until saturation was achieved to ensure reliability, conformability, and mitigation of bias in the delineation of the participant's lived experiences, culture, and faith with regard to hair harvest for routine hospital testing. The study was conducted in line with the consolidated criteria for reporting qualitative studies (COREQ) 15. The areas covered in the interviews were: (i) The cultural meaning of body parts like hair, blood, nails, etc. (ii) If body parts like hair can be of any value or medical meaning. (iii) Perceptions about hospitals starting to use hair for medical testing compared to other samples. (v) Any perceived advantages or disadvantages of using hair for medical testing.

### Data analysis

A thematic analytical approach was adopted with the aid of N-Vivo version 10 software to organize and analyse our data step by step. We initially did data familiarization and writing of familiarization notes. This was then followed by systematic coding and generating initial themes from collated and coded data. Coding was carried out by two independent research nurses with the third independent nurse acting as the tiebreaker in case of code differences between the two main coders to ensure consensus. The independent nurses were not involved in conducting the interview. About 90% of the emerging codes were similar. The principal investigator and the three independent nurses continued developing and reviewing themes, refining, defining, and naming themes until central themes emerged. This step was repeated at least three times until a theme was eventually settled on. This was then followed by writing a report.

### Ethical considerations

This study was approved by the ethics committee of the Makerere University School of Biomedical Sciences (SBS-2021-18) and the Uganda National Council for Science and Technology (HS2231ES). The researchers ensured that all participants were taken through the informed consent, given ample time to think through, consulted were needed, and allowed to make an informed decision whether to participate or not. All consenting participants signed the informed consent form. The researchers ensured participant confidentiality and assured the participants that the findings will be published anonymously.

## Results

Sixteen TB patients participated in the study. The mean age was 33.4(SD=10.2) and gender distribution was equal for male and female participants in the study (Table 2). The results delineate a phenomenon of outpatient TB patients who are already on TB treatment. The content analysis yielded 4 main themes with the first one being participants' perceptions about the cultural meaning of their body parts. The second theme was the medical value or meaning of hair. The third theme was their perceptions of hospitals starting to use hair testing for routine treatment adherence monitoring. The fourth theme was the perceived advantages and disadvantages of using hair for treatment adherence monitoring.

**Table 2:** Characteristics of study participants

Variable	Frequencies	Percentages (%)
Gender		
Male	8	50.00

Age		50.00
18-<30	8	25.00
30-40	4	25.00
>40yrs	4	

Education level		
≤Primary 7	6	37.50
S.1-≤S.4	6	37.50
S.5-S.6	1	6.25
≥S.6	2	12.50
No education	1	6.25

Experience in Hospital testing		
Yes		
No	12	75.00
Other	3	18.75
	1	6.25

Duration of TB symptoms		
0-≤3 weeks		
>3-4weeks	8	50.00
>4 weeks	1	6.25
other	6	37.50
	1	6.25

### Theme 1: Participant's perception of cultural/religious meaning of their body parts

Most participants emphasized the cultural/religious meaning of their body parts like hair attributing it to being an indicator of the completeness of creation by God. They also attributed hair to be a predictor of one's lifestyle.

One participant described

*“These are some parts that make you complete for example if you have hair, you are a complete human being. If you see someone without hair, you can say the person is not complete. Biblically hair is part of the body and shows one is a complete human being”*.

### Theme 2-Hair medical value and meaning

About 94% of the participants described the hair as having specific medical values and meanings.

They described the value of hair as being able to cushion the head and protect it from harsh weather like extreme temperatures. Some participants attributed hair to being useful for DNA testing just like other samples including blood. Some described hair color as a possible indicator of the presence of some disease in the body or lack of certain food substances.

One participant described:

*“Yes I do attribute medical values to it because hair grows on your body depending on what you eat and I think it could contain properties from your blood and shows characteristics of your lifestyle and if you don t eat some nutrients, you end up having thinner than normal hair”*

### Theme 3: Perspective on hospitals starting to use hair for routine treatment adherence monitoring

**Figure 1:**
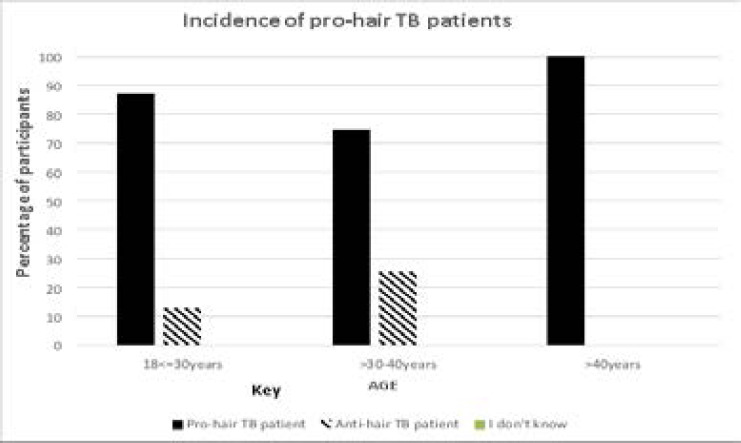


About 88% of the patients interviewed expressed willingness to have their hair used for routine hospital testing only if it is a medical worker carrying out this test (figure 1). They expressed reservations and unwillingness in case it is a none medical person taking their hair for testing. They expressed that hair is one way of performing acts of witchcraft in one's life and caution has to be exercised on whom you give your hair to.

One participant described

*“For hospitals, if a doctor decides to remove hair for a checkup, you have a right to accept because he knows why he's doing it and so I have no problem with that but I don't agree with a none medical person taking my hair; what for?”*

Then one other participant described

*“Using hair is something too much. I have never even heard about it. It is going overboard. Someone takingyour hair can have the intention to harm you in witchcraft?”*

### Theme 4: Perceived advantages and disadvantages of using hair for treatment adherence monitoring

About 81% of the interviewed participants expressed the advantages of using hair for routine medical testing compared to other methods but they hardly expressed any disadvantages. The advantages expressed were for hair is easy to use, and not painful to collect from the body compared to blood where they have to prick someone's body. The disadvantage expressed was using the same equipment to cut hair across different people which carries the risk of infection transmission.

One participant described

*“Advantages: It's faster and less scary than using blood, they just remove a strand of hair and that is it. I have no dis advantages”*

Then another participant described

“If the hair is harvested the way it is cut in salons where they use the same machine to cut different people, one can easily get infected with diseases”

## Discussion

Qualitative research regarding the use of hair harvest and testing to monitor adherence to TB treatment is innovative and important for improving future treatments not only for TB but for other conditions like HIV, epilepsy, etc. The main themes in our study; were the participant's perception of hospitals starting to use hair routinely to monitor adherence to TB treatment, participants' perception of the cultural/religious meaning of their body parts, hair's medical values and meaning, and perceived advantages and disadvantages of using hair for treatment adherence monitoring. The themes emerged from descriptions by participants in the context of their life experiences, culture, and faith.

The first theme identified in our study was perceptions of the cultural/religious meaning of body parts. Most participants emphasized the cultural/religious meaning of their body parts like hair attributing it to being an indicator of the completeness of creation by God. They also attributed hair to be a predictor of one's lifestyle. Hair was noted as a cover of the head that is protective against extreme weather by the majority of the participants. Participants also attributed hair as a medium through which witchcraft can be accomplished by one's enemy. A number of participants described hair harvest as being against their culture especially cutting hair at night. Cotzee et al etal12 did a study on reactions, beliefs, and concerns associated with providing hair specimens for medical research among South African HIV-infected women on antiretroviral therapy. He found that despite knowing that only a few strands of hair were needed for medical testing, the witchcraft concern was outstanding among the study participants. This study is consistent with our study findings. The witchcraft concern found in our study is also consistent with prior beliefs that have been held in the opinion of African society that hair can be used as a medium of witchcraft.

The second theme identified in our study was the medical values and meaning attached to the hair. Many delineated hair as being able to perform a role of DNA identification similar to the role played by blood or other body parts. Grisedale et al 16 did a study on nuclear DNA profiling of rootless hair shafts. The results demonstrated the isolation of nuclear DNA with high precision from rootless hair shafts. This study confirms the perception displayed by our participants that hair can be used for DNA testing.

The third theme identified in our research was the participant's perspective about hospitals starting to use hair for routine hospital testing as a method to monitor adherence to TB treatment. The majority of the participants expressed willingness to have their hair tested in the hospital provided it is a medical worker harvesting the hair from their bodies. They expressed serious reservations and unwillingness to give away their hair if it is a none medical person who is asking for the hair from their bodies. The participant's unwillingness to allow none medical personnel to take hair away from their bodies was the fear of witchcraft. This is consistent with prior beliefs that in the African setting, hair is a sensitive matter, and harvesting hair from one's body can be attributed to witchcraft. In our study, the exception for giving hair to none medical personnel was only for salon hair works where the participants described it as “for salons, this hair is always discarded at the end of the day, which makes it less worrisome”. Herbertson et al.^17^ studied patient willingness to donate hair for biomedical research. He found that in an ethnically diverse, urban-based Nigerian study population; nearly two-thirds of the participants were willing to donate hair samples for biomedical research. Our study findings on this theme are in agreement with the findings by Herbertson et al^17^ and also the findings by Coetzee etal^12^

The fourth theme we identified in our research was the perceived advantages and disadvantages of using hair as a routine hospital test for TB treatment adherence monitoring. Most of the interviewees expressed the advantages of using hair for routine hospital testing like it being easy to harvest, and not being painful like needle pricks would be for blood draws. The majority didn't express any disadvantages but a few had concerns about using the same equipment to cut hair from different people which would increase the risk of skin infections across different patients. Pragst et al.^18^ studied hair samples to investigate alcohol abuse. It was found that the use of hair strongly increases the accuracy of the diagnosis by mutual confirmation and identification of false positive or false negative results due to biological variations or analytical errors. The findings in our study supplement the perceived advantages expressed by Pragst et al in using hair for alcohol abuse testing.

The strength of our study is that it was carried out in the typical traditional African setting where there are lots of cultural beliefs and witchcraft ideologies that have lived within these communities for centuries.

The weakness of our study is that we didn't probe for specific religious affiliations per participant which could confound some of the responses we witnessed in the interviews.

## Conclusions

The main aim of this research was to explore the acceptability of hair harvest as a method of therapeutic drug monitoring among pulmonary TB patients in the context of participant's lived experiences, culture, and religion/faith. The majority of the participants interviewed in this study were okay with using their hair for routine hospital testing to monitor adherence to TB treatment provided the hair was harvested and tested by a medical worker.

### Implications for policy

1. There's a need to develop a plan to sensitize the general public that hair can be used to do routine hospital testing to break the traditional jinx that hair harvested from one's body is meant for witchcraft.
2. Based on our findings, further qualitative research is needed to examine these themes for generalization in order to prepare TB patients and the general patient community for future tests involving hair.
